# Silicon twisted cone structure produced by optical vortex pulse with structure evaluation by radiation hydrodynamic simulation

**DOI:** 10.1038/s41598-020-77323-4

**Published:** 2020-12-01

**Authors:** Daisuke Nakamura, Ryohei Tasaki, Miki Kawamoto, Hiroki Oshima, Mitsuhiro Higashihata, Hiroshi Ikenoue, Toshitaka Wakayama, Atsushi Sunahara, Takeshi Higashiguchi

**Affiliations:** 1 Graduate School of Information Science and Electrical Engineering, Kyushu University, 744 Motooka, Nishi-ku, Fukuoka, 819-0395 Japan; 2 Saitama Medical University, 1397-1 Yamane, Hidaka, Saitama 350-1241 Japan; 3 Center for Materials Under Extreme Environments (CMUXE), School of Nuclear Engineering, Purdue University, West Lafayette, IN 47907 USA; 4 Institute of Laser Engineering, Osaka University, 2-6 Yamadaoka, Suita, Osaka 565-0871 Japan; 5 Department of Electrical and Electronic Engineering, Faculty of Engineering, Utsunomiya University, 7-1-2 Yoto, Utsunomiya, Tochigi 321-8585 Japan

**Keywords:** Laser material processing, Laser-produced plasmas

## Abstract

We demonstrate a radiation hydrodynamic simulation of optical vortex pulse-ablated microcone structures on silicon (Si) substrates. Doughnut-shaped craters were formed by single pulse irradiation on the Si substrate, and a twisted cone structure with a height of 3.5 µm was created at the center of the irradiation spot by the circularly polarized optical vortex pulse. A two-dimensional (2-D) radiation hydrodynamic simulation reproduced the cone structure well with a height of 3 µm. The central part of the incident laser power was lowered from the initial profile due to plasma shielding over the laser pulse duration for an inverted double-well laser profile. The acute tip shape of the silicon surface can survive over the laser irradiation period.

## Introduction

A transparent microparticle behaves as an optical microcavity providing very high light confinement^1^. Whispering gallery mode (WGM) microcavities, in which light is confined in circular orbits by total internal reflection, have extremely high *Q* factor values. These microcavities can be used in label-free biosensors^2^ and optical couplers^3^ as well as other devices. Generally, glass and polymer materials are used for microcavities because they exhibit visible transparency and are easy to fabricate. However, transparent microparticles require light injection from outside by evanescent coupling using an angle polished fiber^4^. In our research, we have successfully fabricated semiconductor microparticles by a simple laser ablation method in air^5–8^, and have demonstrated ultraviolet WGM lasing from photo-excited single ZnO microparticles without light injection.

There are several laser-based particle fabrication techniques that allow precise control of size and ejection direction. One example is 2D and 3D patterning of metal microparticles based on laser-induced forward (or backward) transfer (LIFT) of molten metal nanodroplets ejected from thin donor metal films towards a receiver substrate^9^. On-demand patterning of oxide microdots by laser irradiation to a source film has also been reported^10^. In addition, Si microdroplet ejection perpendicular to a bulk Si target surface using an optical vortex beam with a doughnut profile has been reported^11,12^, and its mechanism is probably based on laser-driven pressure. Furthermore, in an interesting result, the creation of twisted structures has been reported, corresponding with the total angular momentum of the optical vortex^13,14^. As a twisting force will enhance straight ejection of droplets by the gyro effect, an optical vortex beam is expected to offer a well-controlled fabrication technique for microparticles. In optical vortex irradiation, the laser parameters, such as power density, irradiation spot size, polarization, and ellipticity of the focused beam on the target will be important for understanding the detailed mechanisms of droplet ejection.

There have been many similar reports about the optical vortex laser, matter interaction and ablated structures in many different elements. The laser ablation of Si targets with vortex laser pulses has been reported in several papers, including the formation of similarly twisted protrusions^15,16^. Most of the reports only discuss the light mode and ablated structure as the final state. There have been no reports on the material dynamics and detailed plasma parameters during ns-laser pulse irradiation. The current study is focused on the energy transfer in the laser and matter interaction, reaching the ablated cone structure through a ring pattern focal beam. The final state of the ablated structure resulted from hydrodynamic motion under a spatiotemporal change of the parameters of electron temperature, electron density, and plasma pressure. The structure is formed by the plasma pressure, which is defined by the electron temperature and electron density. It is important to understand the detailed dynamics of the ablated structure during the laser pulse irradiation and final state of the structure. In the present study, we applied a radiation hydrodynamic simulation to evaluate the spatiotemporal behaviors of the plasma parameters, electron temperature, electron density, and plasma pressure, in the ablated plasma.

In this paper, we first demonstrate the twisted cone structure on the Si substrate produced by a polarization state-determined optical vortex pulse with circular polarization. The polarization distribution and intensity profile of the focused optical vortex beam at different defocus positions are visualized. It is found that doughnut-shaped craters were formed by single pulse irradiation on the Si substrate. A radiation hydrodynamic simulation is carried out to consider the ablation mechanism.

## Results

### Optical vortex beam irradiation of Si target

**Figure 1:**
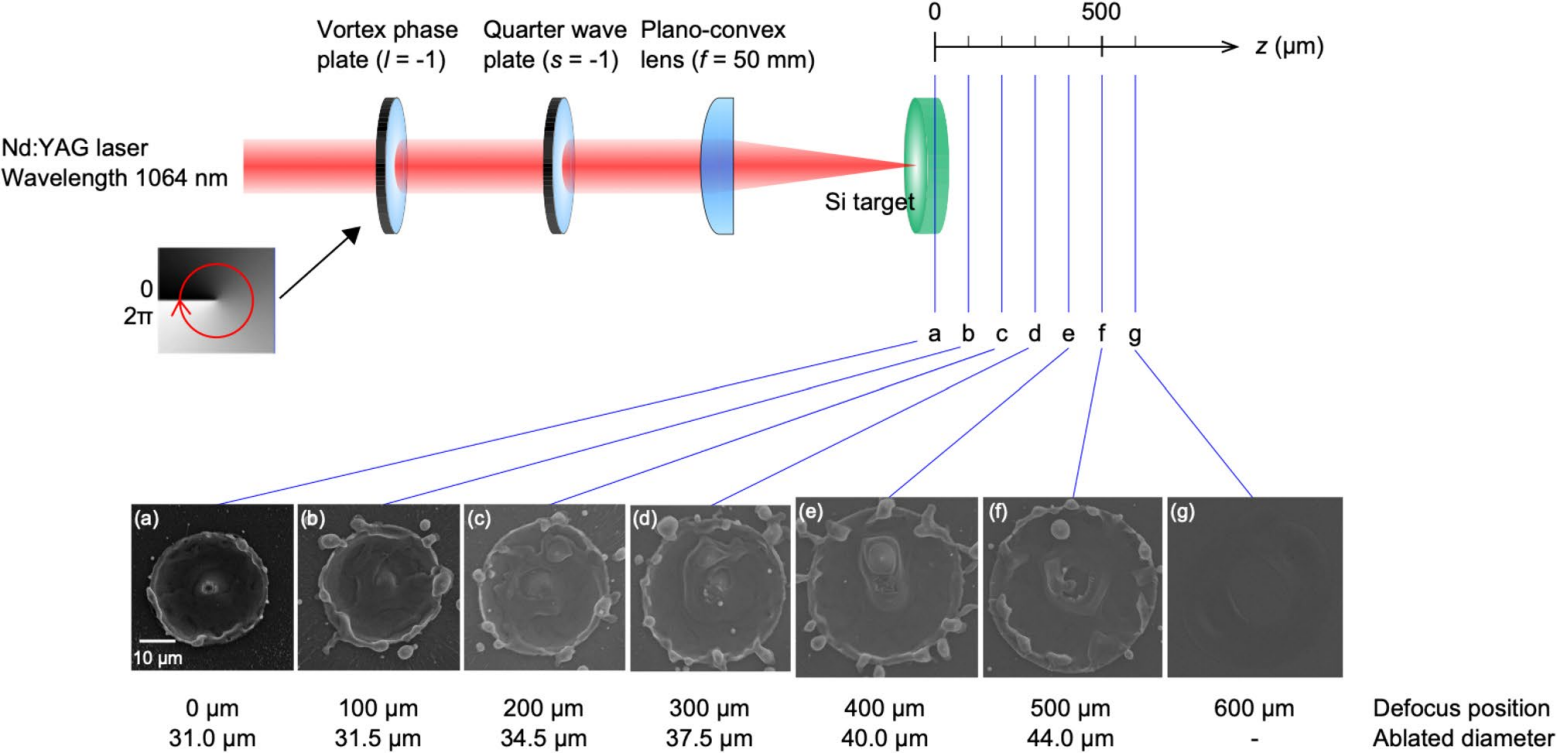
Schematic diagram of the experimental setup of optical vortex beam irradiation and the SEM images of the ablated structures at different target positions.

**Figure 2:**
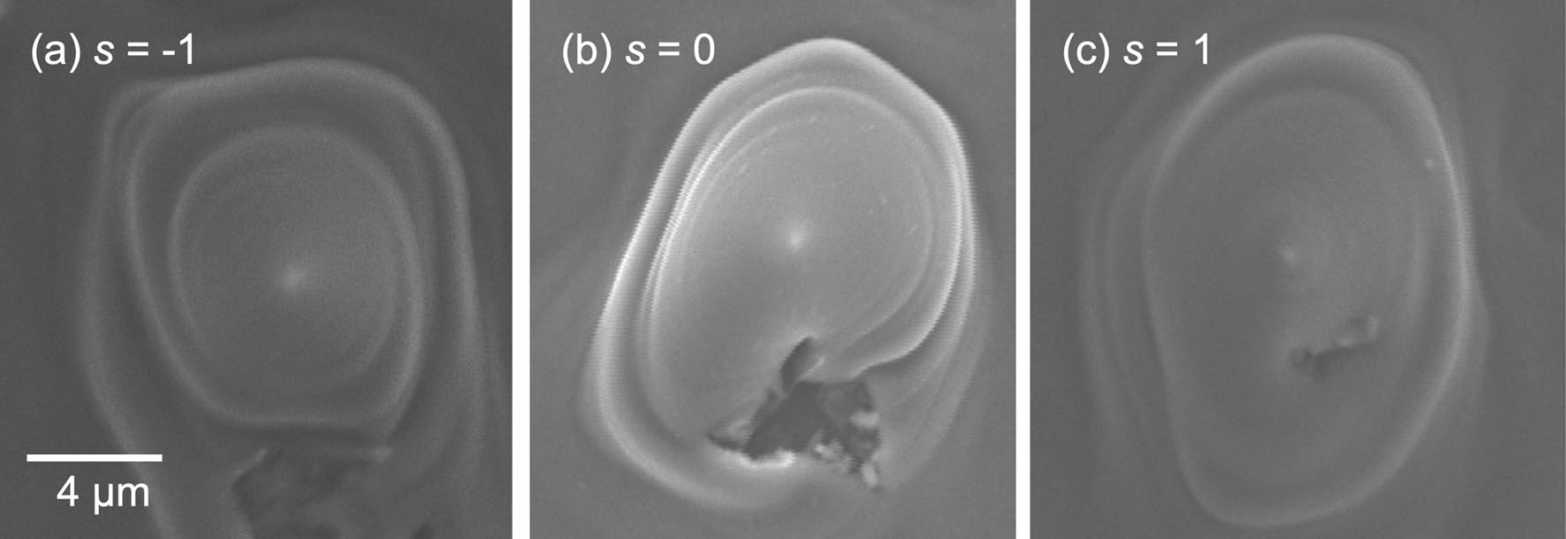
Magnified SEM images of the cone structures at the center of the irradiation spot with (**a**) *s* =  − 1, (**b**) *s* = 0, and (**c**) *s* =  + 1.

We produced a twisted cone structure on a Si substrate irradiated by a polarization state-determined optical vortex pulse. We focused on the polarization-determined optical vortex of the laser ablated target surface because of the importance of quality assurance of the laser ablation. In this experiment, the polarization distribution of the optical vortex beam was determined to be almost uniform as a circularly polarized beam at the focusing area, as shown in Figs. 10 and 11 in “Methods”. However, the azimuthal angle of the elliptically polarized beam near the circular polarization exhibited considerable variety. Figure 1 shows a schematic diagram of the experimental setup. The structures in the images (a)–(g) were probably formed by melting of the target pushed by laser-driven pressure. Interestingly, at the 400-µm defocus position, Fig. 1e, a twisted structure is clearly observed, as shown in Fig. 2a. A twisted microcone structure was formed by the balance between the melting volume and coagulation process, with optimum plasma parameters, such as the plasma pressure and temperature, related to the laser pulse absorption. In the case of the laser irradiation points (a)–(d) in Fig. 1, a periodic structure on the microcone surface is expected to disappear due to the melting after the structure is formed. On the other hand, no surface structure forms at points (f) and (g) because of the lower laser fluence. Note that we observed a small protrusion but no twisted structure at point (f).

**Figure 3:**
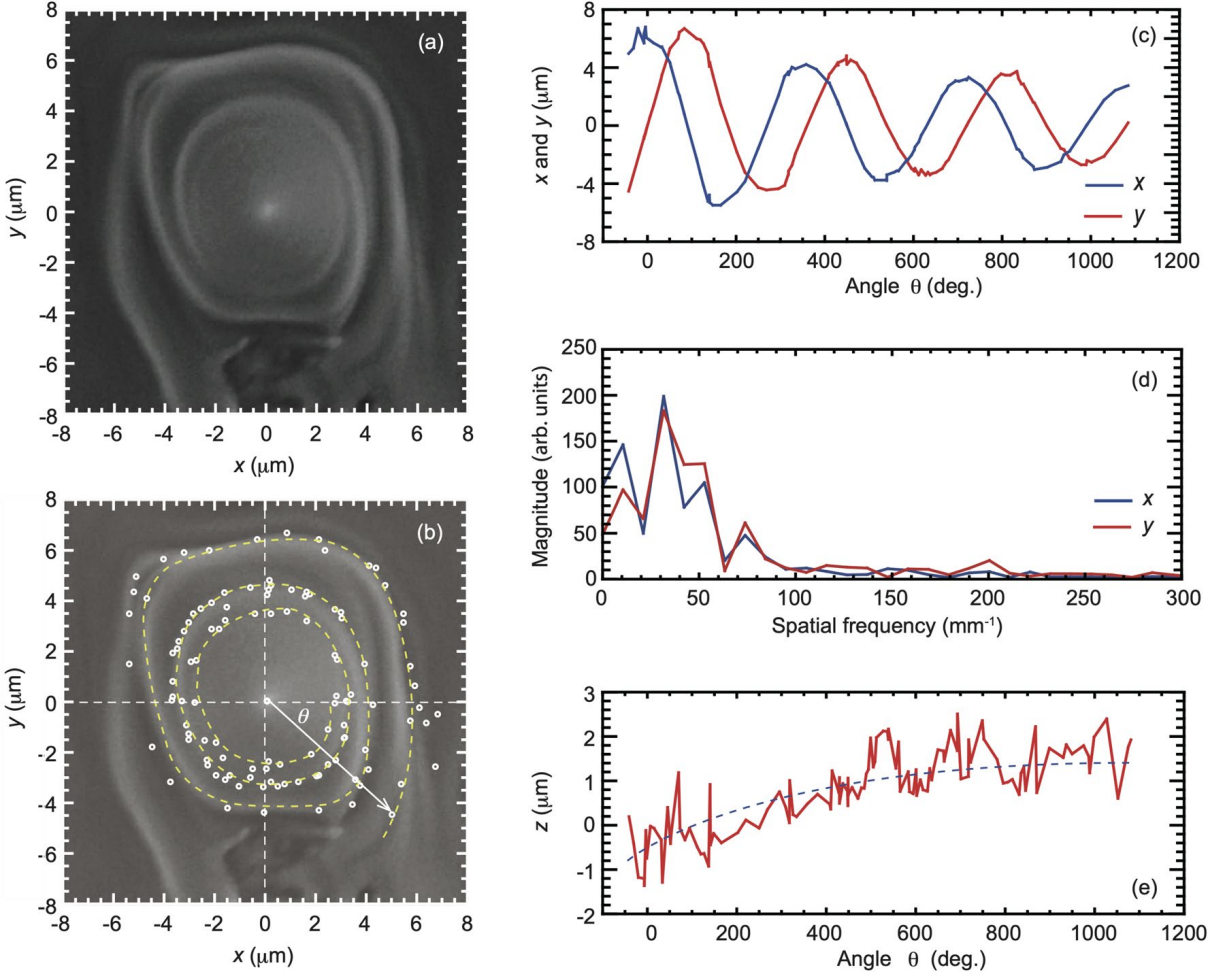
Three-dimensional analysis of an ablated microstructure. (**a**) Enlarged view of Fig. 2a. (**b**) Matching points (white circles) and the spiral path (yellow broken line), drawn on the SEM image. (**c**) *x*- and *y*-coordinates as a function of angle *θ*, showing three laps around the spiral path. (**d**) Spatial frequency distribution determined from the distances between each point (**b**). (**e**) Height on the spiral path.

The cone structures showed twisted microcones displaced with respect to the center of the irradiation spot. We believe that the center of the focal beam was displaced, based on the focal spot pattern at point D in Figs. 10 and 11 in “Methods” due to inaccuracy in the spiral plate. The total angular momentum is described as *j* = *s* + *ℓ*, where *s* and *ℓ* are an integer of a spin and an orbital angular momentum. The twisted structure is expected to be produced by a large total angular momentum, which corresponds to a large rotational force. The rotation direction of the twisted microcone structure is determined by the sign of the total angular momentum of the optical vortex beam. In the present case with an integer constant of *ℓ* =  − 1 in Fig. 2, the total angular momenta were *j* =  − 2 (*s* =  − 1, *ℓ* =  − 1) in Fig. 2a, *j* =  − 1 (*s* = 0, *ℓ*  =  − 1) in Fig. 2b, and *j* = 0 (*s* = 1, *ℓ*  =  − 1) in Fig. 2c. While a twisted structure was observed in Fig. 2a, the structures showed less twisting for small *j* in Fig. 2b,c, with Fig. 2b showing an unclear twisted structure. These results roughly correspond with the previous report^14^. We measured the height of the twisted cone structure using atomic force microscopy (AFM). The height was measured to be + 3.5 µm (not shown), which is in good agreement with the proposed three-dimensional analysis of the microstructure (see below in Figs. 3e and 12g in “Methods”). We also evaluated the height of the microstructure by a radiation hydrodynamic simulation.

### Three-dimensional analysis of the microstructure

We applied three-dimensional analysis to clarify the details of the microstructure obtained by the vortex laser processing. Figure 3a shows an enlarged view of a microstructure with a twisted cone after contrast-adjusting due to analyze the twisted cone. In Fig. 3b, we have overlapped matching points (white circles) on the SEM image. The matching points trace the twisted cone path of three turns illustrated by the yellow dashed line. Figure 3c shows *x*- (blue line) and *y*-coordinates (red line) on the twisted cone path. The *x-* and *y*-coordinates form trigonometric functions of the angle *θ*. We performed a Fourier analysis of the distances between each point in Fig. 3b to determine the spatial frequency of the twisted cone. The length of the periodic structure was 30 µm, as seen in Fig. 3d. Note that the radius of the periodic structure changes. Figure 3e shows the height on the twisted cone path, ranging from *z* =  − 1.0 to 1.5 µm. The twisted cone path disappeared at the heights *z* = 1.5 to 3.6 µm and changed to a cone shape.

### Simulation and discussion

**Figure 4:**
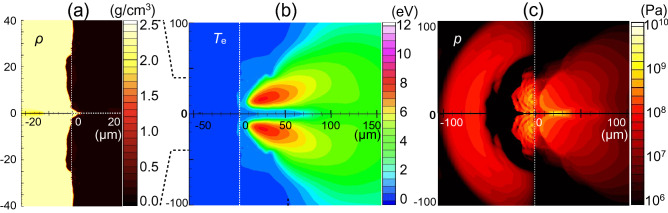
Calculated profile at *t* = 20 ns with an inverted double-well laser profile condition. The spatial scale is in µm. (**a**) Density profile (g/cm^3^), (**b**) electron temperature *T*_e_ (eV), and (**c**) pressure *p* (Pa). The broken lines represent the initial surface of the silicon (*z* = 0).

Calculated profiles at a laser peak time of *t* = 20 ns are shown in Fig. 4a–c. The density profile of the laser-irradiated Si shows a shallow crater with an acute tip along the *z*-axis in Fig. 4a. The ablated silicon plasma expanding to the vacuum is heated to 8 eV, except for the slender region up to *z* = 70 µm along the *z*-axis which has a relatively low temperature of 2 eV, as shown in Fig. 4b. The pressure profile in Fig. 4c shows two characteristic patterns, the leading hemisphere pressure peak and the largest peak spot at the irradiated Si surface up to 0.5 GPa, in the silicon bulk. This peak pressure is consistent with a pressure *p* of 0.5% compressed Si, estimated from the bulk modulus, *K*, defined as follows:

1 $$K = - V\left( {\frac{\partial p}{{\partial V}}} \right)_{T} ,$$

where *V* and *T* are the specific volume of silicon, defined as *V* = 1/ρ, and the temperature, respectively. The bulk modulus *K* of silicon is 98 GPa^17^. The density of the compressed region at the irradiated silicon surface is 2.32 g/cm^3^, roughly 0.5% compression (|∆*V*|/*V* ≈ 0.5%) from the initial density. Thus, the pressure is calculated to be *K*|∆*V*|/*V*.

**Figure 5:**
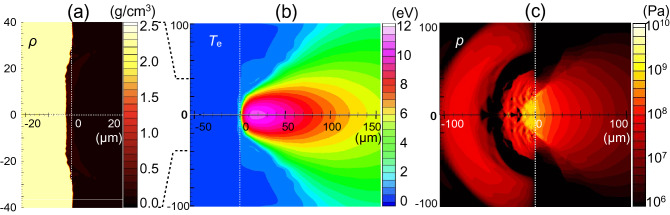
Calculated profile at *t* = 20 ns with a Gaussian laser profile condition. The spatial scale is in µm. (**a**) Density profile *ρ* (g/cm^3^), (**b**) electron temperature *T*_e_ (eV), and (**c**) pressure *p* (Pa). The broken lines represent the initial surface of the silicon (*z* = 0).

To compare these calculated profiles with those for a conventional Gaussian laser spatial profile, we carried out a simulation with the parameters *A*_1_, *A*_2_, and ∆*R* in Eq. (5) in “Methods” set to 4.3 × 10^9^ W/cm^2^, 0.0, and 15 µm, respectively. The laser temporal profile and total energy are unchanged. Calculated profiles are shown in Fig. 5a–c. Under the Gaussian condition, the density profile of laser-irradiated silicon shows a shallow crater with a flat bottom, and the ablated plasma plume is heated to 10.5 eV. The pressure applied to the silicon bulk in Fig. 5c shows two hemisphere profiles, similar to that in Fig. 4c.

**Figure 6:**
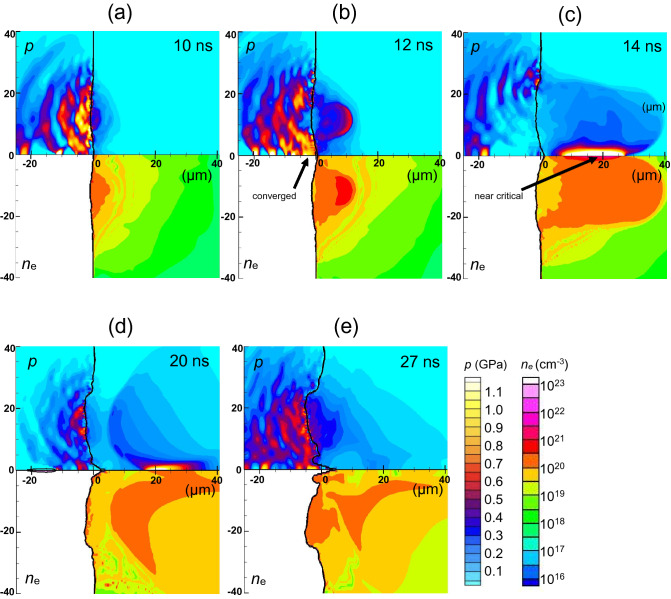
Temporal evolution of the distributions of the pressure and the electron density due to the inverted double-well intensity profile laser pulse irradiation at times of *t* = 10 (**a**), 12 (**b**), 14 (**c**), 20 (**d**), and 27 ns (**e**).

From a comparison between Figs. 4a and 5a, we inferred that the acute tip shape of the laser-irradiated silicon surface along the *z*-axis in Fig. 4a can be attributed to the inverted double-well laser spatial profile. To understand characteristic profiles with the inverted double-well laser profile condition, we show the pressure and electron number density profiles at five different timings in the range *t* = 10–27 ns, as shown in Fig. 6. In Fig. 6a, we see that a large amount of ablated plasma is generated around *r* = ± 14 µm, corresponding to the intense part of the inverted double-well laser profile. The pressure applied to the Si bulk propagates spherically, and part of the pressure wave converges at the z-axis and generates a high-pressure region near the surface, as shown in Fig. 6b. This high pressure drives the Si in the positive *z*-direction. Then, the surface of the silicon around *r* = 0 starts to lift above the initial position of the silicon surface, *z* = 0. At *t* = 14 ns in Fig. 6c, ablated plasma plumes collide with each other at the *z*-axis and generate a high density region, in which the electron number density is high, though slightly lower than the critical density. This region can absorb the laser energy incoming from the right simulation boundary, decreasing the laser power arriving at the silicon surface. Consequently, the laser absorption power density at the critical density close to the silicon surface is reduced and modulated from the initial incident laser power density *I*_*L*_.

**Figure 7:**
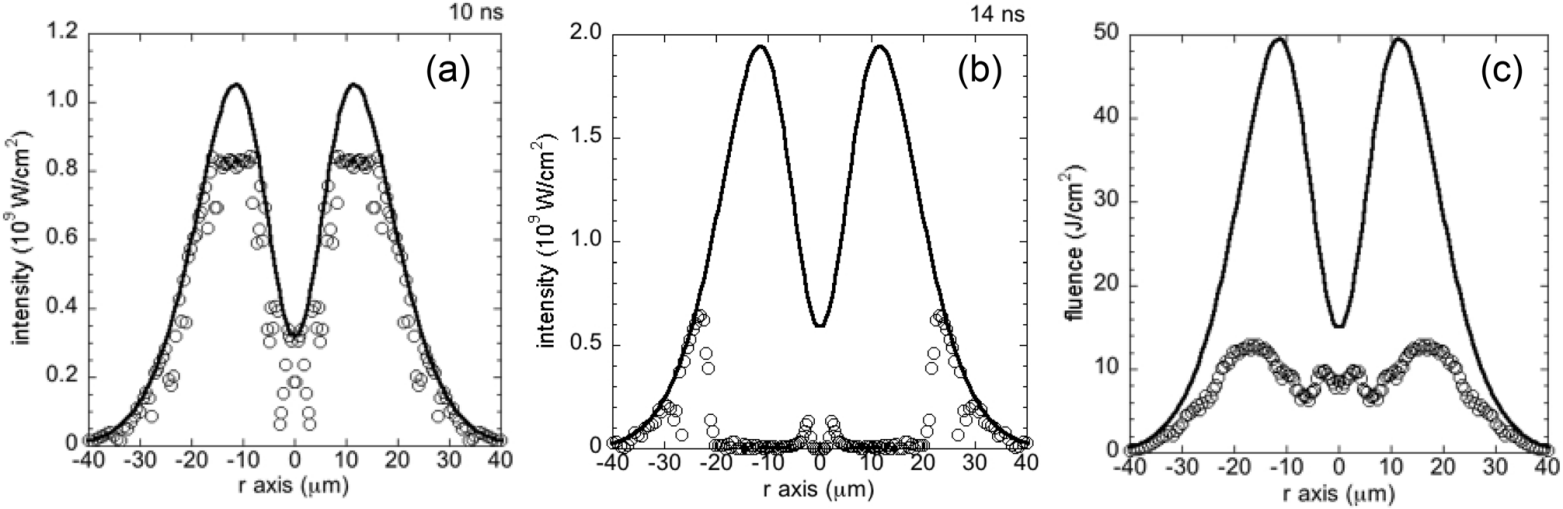
Initial power density of the incident laser at the boundary of the simulation box (solid line) and the calculated absorbed power density at the Si surface along the *r*-direction (circles), for the inverted double-well laser profile condition, (**a**) at *t* = 10 ns in W/cm^2^, (**b**) at *t* = 14 ns in W/cm^2^, and (**c**) time integrated over the laser pulse (fluence) in J/cm^2^.

To confirm this modulation of the laser power arriving at the silicon surface due to the ablated plasma plume, we show the incident laser power density and the absorbed power density at *t* = 10 ns in Fig. 7a and 14 ns in Fig. 7b. The differences between the incident and absorbed power density profiles show that the laser energy is absorbed by the ablated plasma plume on the way to the surface of the bulk silicon. Figure 7a shows that most of the incident laser energy is absorbed at the silicon surface at *t* = 10 ns. The amount of ablated silicon increases in time, and thus the laser power density is lowered due to the plasma shielding of the laser at *t* = 14 ns, as shown in Fig. 7b. At this time, the absorbed laser power density is highly modulated from the initial profile. We see that most of the laser power near the *z*-axis (*r* = 0) is lost at *t* = 14 ns, and this extreme modulation of the absorbed laser power density around *r* = 0 corresponds to the high density region produced by the collision of the ablated plasma near the *z*-axis shown in Fig. 6c. This plasma shielding of the laser power can keep the plasma region in front of the low temperature and low pressure silicon surface, as shown in Fig. 4b,c. After the laser intensity peak, the pressure and the electron density decrease due to a lowering of the electron temperature, as shown in Fig. 6d,e. Accordingly, the plasma shielding reduces. However, the plasma shielding can modulate the absorbed laser fluence at the silicon surface from the incident profile, as shown in Fig. 7c. We conclude that the central part of the incident laser power is lowered from the initial profile due to the ablated plasma shielding over the laser pulse duration in the inverted double-well laser profile case. Thus, the acute tip shape of the silicon surface can survive over the laser irradiation period.

**Figure 8:**
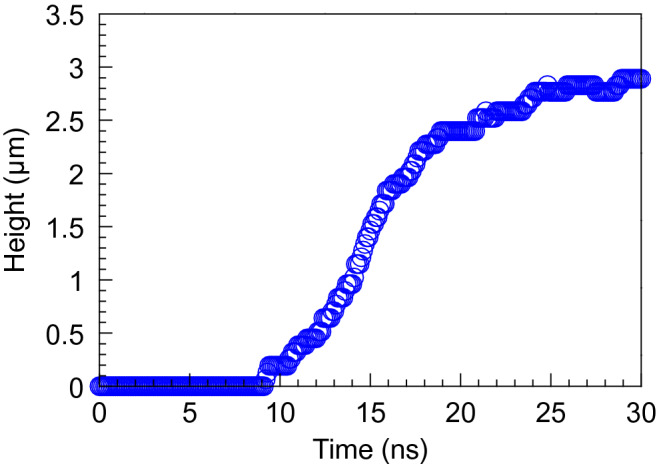
Calculated temporal evolution of the height of the cone structure on the Si substrate.

Figure 8 shows the calculated temporal evolution of the height of the cone structure on the Si substrate. The height of the cone structure increased with time, and the height is saturated at around 3 µm. The simulation result reproduces the observed twisted cone height of 3.5 µm in the present experiments and analysis.

## Summary

In summary, we have described the ablated structures on Si substrates irradiated by polarization-determined optical vortex laser pulses and using the radiation hydrodynamic simulation. The polarization distribution and intensity profile of the focused optical vortex beam at different defocus positions were experimentally visualized using a rotating quarter wave plate and a polarizer. Doughnut-shaped craters were formed by single pulse irradiation on the Si substrate, and a twisted cone structure with a height of 3.5 µm was created at the center of the irradiation spot by the circularly polarized optical vortex pulse. The 2-D radiation hydrodynamic simulation reproduced the cone structure with a height of 3 µm. We concluded that the central part of the incident laser power was lowered from the initial profile due to the plasma shielding over the laser pulse duration for the inverted double-well laser profile case. Thus, the acute tip shape of the silicon surface can survive over the laser irradiation period. We believe that our results can be extended to enhance straight ejection of droplets by the gyro effect. Also, optical vortex beams are expected to be employed as a well-controlled fabrication technique of microparticles.

## Methods

### Determination of polarization state

**Figure 9:**
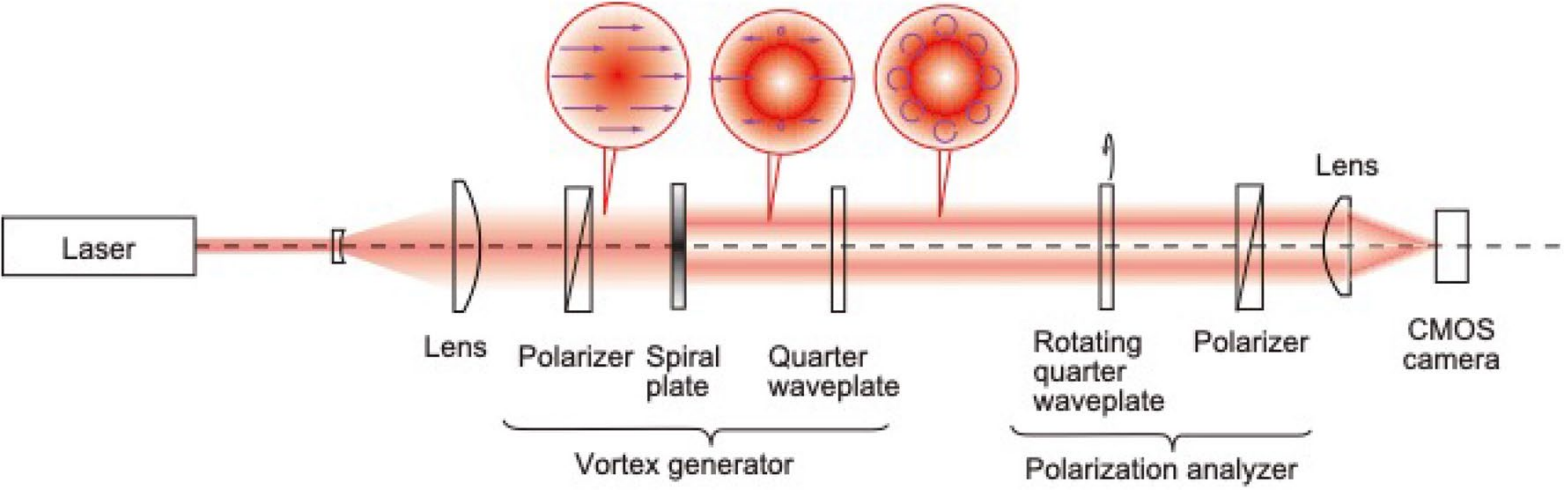
Experimental apparatus for optical vortex beams with circular polarization.

We determined the polarization states of the converted optical vortex nanosecond pulse. Figure 9 shows the experimental apparatus for the generation and polarization analysis for the optical vortex beam processing. A laser is employed as a light source. After passing through a polarizer and two lenses, the laser beam with horizontal linear polarization is incident on the vortex beam generator. Figure 9 shows the experimental apparatus for the optical vortex beam generation and the determination of the polarization state. The vortex beam generator consists of a linear polarizer and a vortex phase plate (RPC Photonics, VPP-m1064), which is fabricated by gray scale lithography. A Gaussian beam with a uniform phase is converted into a Laguerre-Gaussian beam, like a doughnut-shaped beam. The Laguerre–Gaussian beam possesses a phase singularity with the vortex phase. The phase singularity beam is also called the “optical vortex beam.” The optical vortex beam has an orbital angular momentum with *ℓ* =  + 1, where  *ℓ* is an integer. An optical vortex beam with circular polarization is generated by passing through a quarter wave plate. The quarter wave plate enables us to generate a spin angular momentum with *s* =  + 1, where *s* is also an integer. This optical arrangement enables us to manipulate a total angular momentum *j* = *s* +  *ℓ*. To obtain the focusing beam quality, we determined the polarization distribution using a polarization analyzer, which consists of a rotating quarter wave plate and a polarizer^18–20^. The intensity distribution of the vortex beam along the optical axis *z* is recorded by a two-dimensional detector.

**Figure 10:**
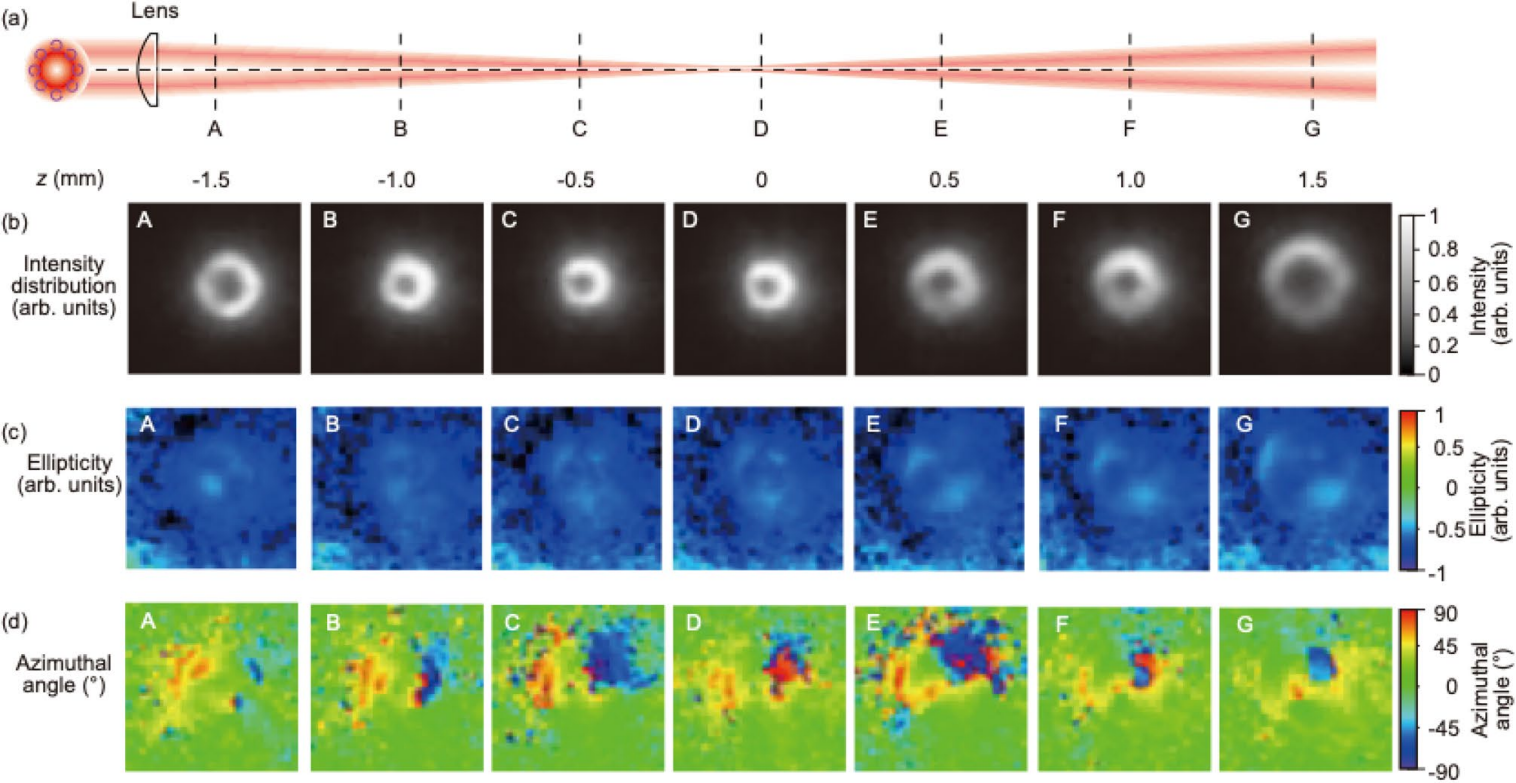
Focusing polarization properties of optical vortex beam with circular polarization. (**a**) Measurement position of CCD camera detector. (**b**) Intensity distributions, (**c**) ellipticity, and (**d**) azimuthal angle measured at positions A to G. Note that the ellipse on the intensity describes the polarization profile of the optical vortex with circular polarization.

**Figure 11:**
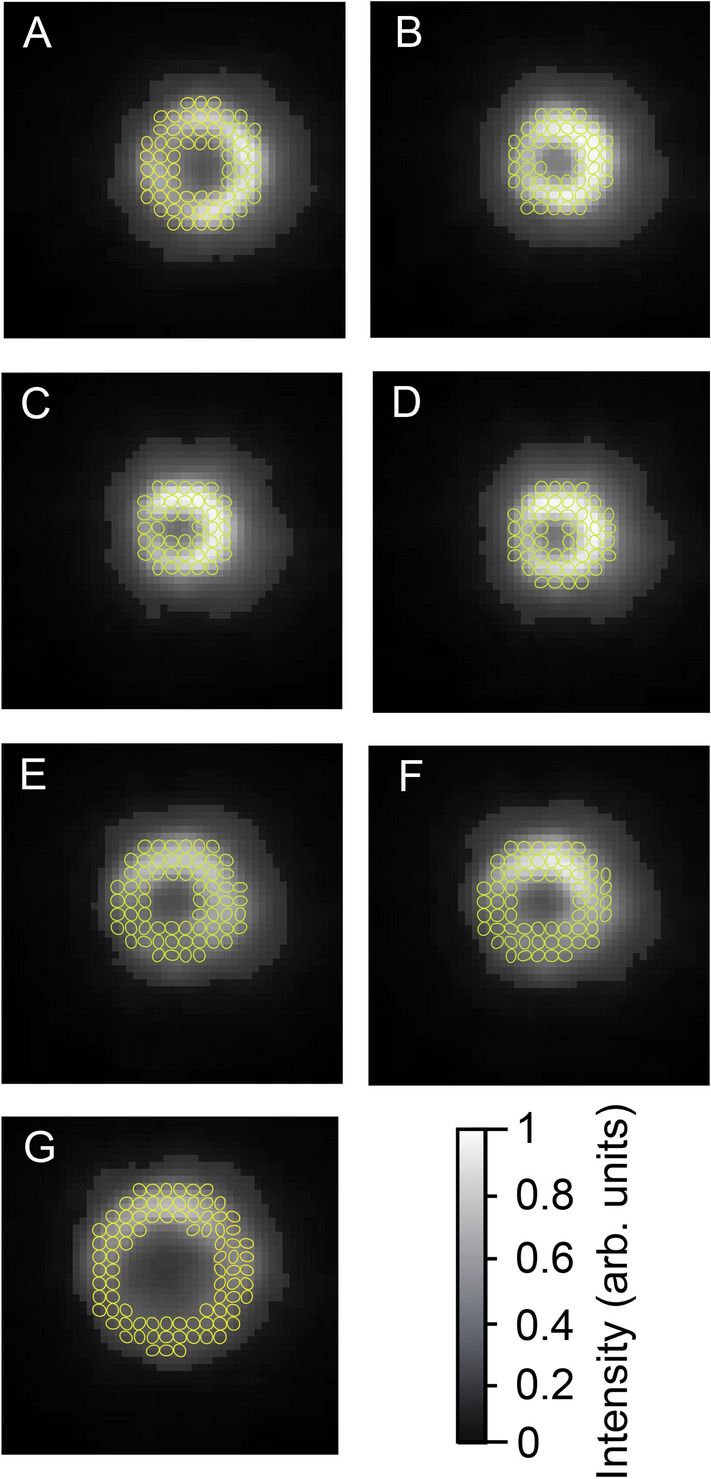
Ellipticity maps of the optical vortex beam measured at positions A to G in Fig. 2a.

In addition to an optical vortex beam with linear polarization, we evaluated the optical vortex with circular polarization to obtain the total angular momentum *j* = *s* +  *ℓ*. Measurements were also taken at positions A to G to examine the focusing polarization property of the optical vortex beam for laser processing in Fig. 10a. The distributions of intensity, ellipticity, and azimuthal angle are also depicted in Fig. 10b–d, respectively. The ellipticity became *ε* =  − 0.85. Interestingly, the azimuthal angle changed axially symmetrically. For the ellipticity and the azimuthal angle, we also calculated the polarization shape. This gave an elliptical polarization near circular polarization, as shown in Fig. 11. The polarization shape was disturbed in the dark area of the optical vortex beam. The polarization analysis helps us to visualize the focusing polarization property of the optical vortex beam.

### Experiments

Figure 1 shows the experimental setup for the irradiation of an optical vortex beam on a Si target. A *Q*-switched Nd:YAG laser (Spectra Physics, Quanta-Ray, LAB-150) with a wavelength of 1064 nm and a pulse duration of 17 ns was used as the ablation laser. A linearly polarized Gaussian beam from the laser was converted into a circularly optical vortex beam through the vortex phase plate with *ℓ* =  − 1 and the quarter wave plate (QWP), as shown in Fig. 1. A single pulse of the doughnut beam was focused by a plano-convex lens (*f* = 50 mm) on the surface of the Si target. The irradiation spot size and laser fluence are key factors for the droplet ejection. Thus, in this experiment, the spot size was changed by increasing the distance between the Si target and the focus lens from the focal point (*z* = 0 µm) to *z* = 600 µm at 100 µm intervals. The pulse energy was fixed at 0.08 mJ. The irradiated surface was observed by a scanning electron microscope (KEYENCE, VE7800). The ablated structures were gradually increased with defocusing, as shown in Fig. 1. Cone structures were formed at the center of the irradiation spots at *z* = 0–400 µm.

### Quantitative three-dimensional analysis

**Figure 12:**
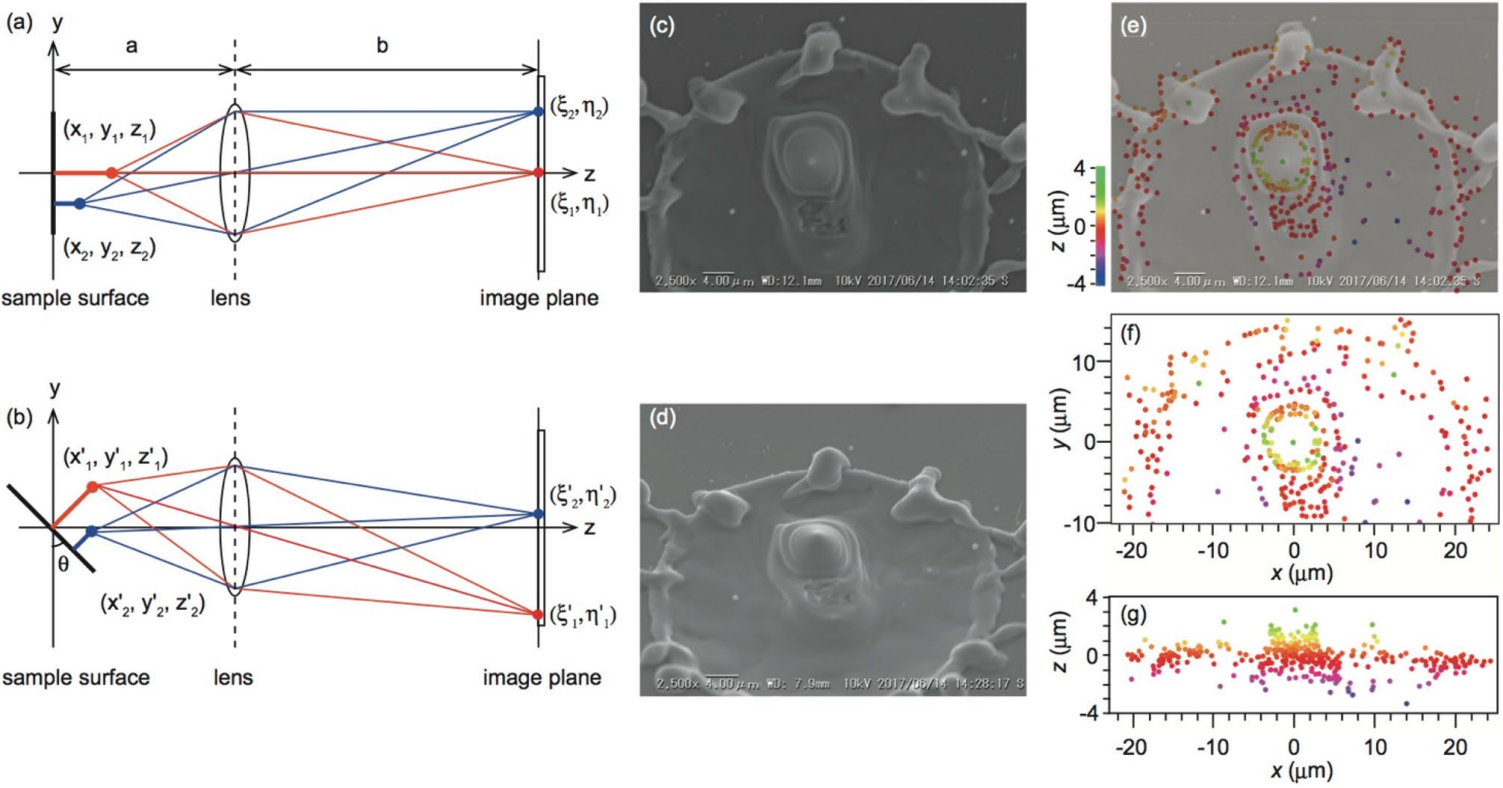
Three-dimensional reconstruction by stereo SEM. (**a**) Optical alignment and (**b**) SEM images at a viewing angle of 0º. (**c**) Optical alignment and (**d**) SEM image at 45º. (**e**) Height map overlapped on (**c**). (**f**) Top view and (**g**) side view of the height map.

Scanning electron microscopy (SEM) and/or atomic force microscopy (AFM) are generally carried out to measure microstructures. The height was measured to be + 3.1 µm using the laser microscope in Fig. 12a. No structural trace is shown here. We applied quantitative three-dimensional analysis to evaluate the micro-structure surface fabricated by the above-mentioned laser processing. Two SEM images captured at two different viewing angles enable quantitative three-dimensional analysis based on a stereoscopic SEM measurement^21–23^. Figure 12a,b show the optical arrangements of the stereo scanning microscope. The electron is incident on the sample surface set at 0º in Fig. 12a. A secondary electron detector captures the SEM image. Arbitrary positions (*x*_1_, *y*_1_, *z*_1_) and (*x*_2_, *y*_2_, *z*_2_) are imaged at points ($\xi_{1}$, $\eta_{1}$) and ($\xi_{2}$, $\eta_{2}$) on the image plane, respectively. Then, we captured an image at another viewing angle after setting the sample surface at angle *θ*, as shown in Fig. 12b. The arbitrary positions ($x_{1}^{\prime}$, $y_{1}^{\prime}$, $z_{1}^{\prime}$) and ($x_{2}^{\prime}$, $y_{2}^{\prime}$, $z_{2}^{\prime}$) were also imaged at points ($\xi_{1}^{\prime}$, $\eta_{1}^{\prime}$) and ($\xi_{2}^{\prime}$, $\eta_{2}^{\prime}$) on the image plane, respectively. A triangulation method enables us to determine arbitrary point cloud data of (*x*_*i*_ , *y*_*i*_ , *z*_*i*_) on the sample surface. Using a point ($\xi_{i}^{\prime}$, $\eta_{i}^{\prime}$) on the image plane, the point cloud data can be expressed as

2 $$x_{i} = \frac{{\xi_{i} }}{m},$$

3 $$y_{i} = \frac{{a - z_{i} }}{ma}\eta_{i} ,$$

4 $$z_{i} = \frac{{\frac{{ma^{2} \eta_{i} \cos \theta + a\eta_{i} \eta_{i}^{\prime} \sin \theta }}{ma} - a\eta_{i}^{\prime} }}{{\frac{{\eta_{i} \left( {ma\cos \theta + \eta_{i}^{\prime} \sin \theta } \right)}}{ma} - \left( {ma\sin \theta + \eta_{i}^{\prime} \cos \theta } \right)}},$$

where *a* and *b* are lengths from the sample to the lens and from the lens to the image plane, respectively, and *m* is the magnitude of the scanning electron microscope.

For the three-dimensional reconstruction of the scanning microscope, we conducted a three-dimensional analysis of the fabricated structure. Figure 12c,d show SEM images at viewing angles of *θ* = 0º and *θ* = 45º, respectively. From their stereo images, we manually matched 372 distinctive points, because the images are limited by automated stereo matching. The heights at the distinctive 372 points were determined, as shown in Fig. 12e. The height was overlapped on the top view shown by Fig. 12c, and illustrated by a color scale. Figure 12f,g are the top and side views of the reconstructed height distribution, respectively. The maximum height of the laser processing was + 3.6 µm on the top of the protrusion, close to the value of + 3.1 µm measured by the laser microscope. The maximum depth by the laser processing was − 3.9 µm.

### Radiation hydrodynamic simulation

**Figure 13:**
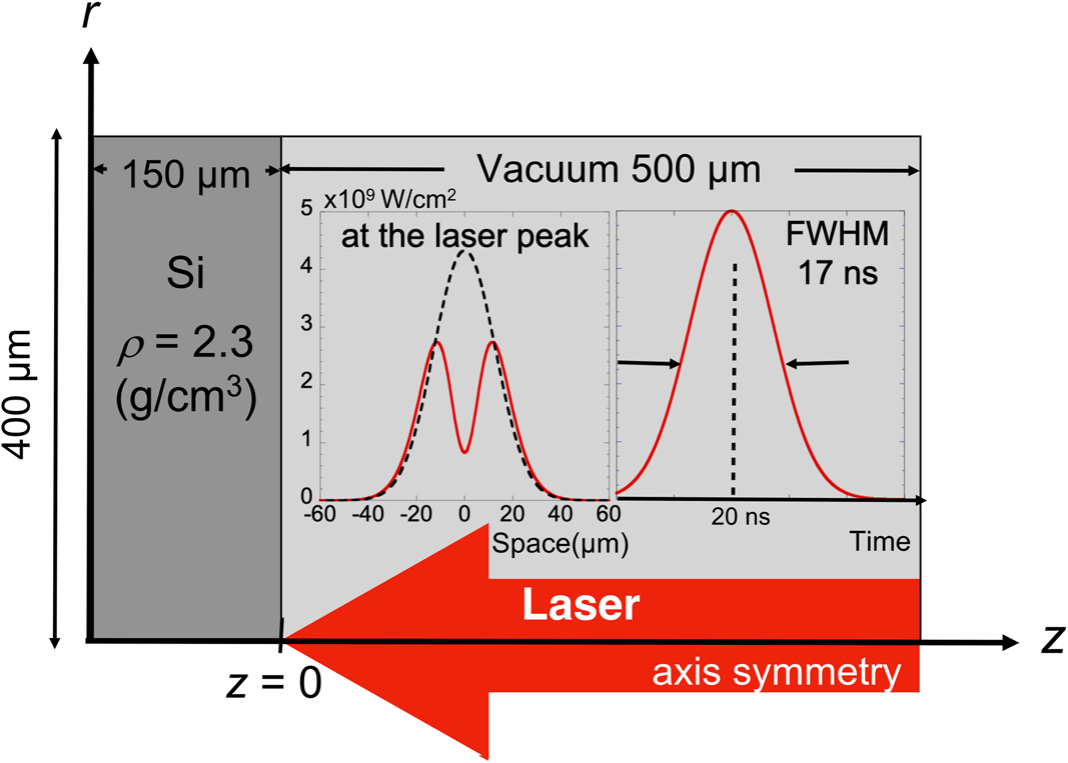
Schematic diagram of the simulation condition.

To simulate the laser ablation driven by a laser having an inverted double-well intensity profile, we carried out a two-dimensional radiation hydrodynamic simulation using the STAR-2D code, which solves hydrodynamics problems, accompanied by energy transfer processes such as laser propagation, thermal conduction of electrons and ions, and radiative energy transport^24^. As a realistic equation of state (EOS) for Si, the SESAME data table was adopted^25^. In the simulation, the laser absorption is treated as inverse-bremsstrahlung^26^, and the radiative energy transfer is described by the flux-limited multi-group diffusion approximation model with 40 bins of radiative emissivity and opacity, ranging from 0 to 1 keV photon energy, calculated by the collisional radiative steady-state (CRSS) model^27^. As shown in Fig. 13, the simulation region was set to have cylindrical geometry (*z*, *r*) with a size of 650 µm in the *z*-direction and 400 µm in the *r*-direction, and unequal zoning meshes of 350 × 150 were assigned to resolve the sharp density gradient of laser ablation on the silicon surface. A 1064-nm laser pulse was injected normal to the surface of the silicon bulk (*z* ≤ 0) with a density of 2.31 g/cm^3^, along the z-axis, through the low-density region (*z* > 0) with 10^−7^ g/cm^3^ initial density, mocking up the vacuum. The laser temporal profile is Gaussian with a pulse duration of 17 ns (FWHM), having a peak intensity of 2.75 × 10^9^ W/cm^2^ at the simulation time *t* = 20 ns. The pulse energy is 0.8 mJ for the present laser parameters. The laser spatial intensity profile is shown in Fig. 13, given by

5 $$I_{L} (r) = A_{1} \left\{ {\exp \left[ { - \ln 2\left( {\frac{r}{\Delta R}} \right)^{2} } \right] - A_{2} \exp \left[ { - \ln 2\left( {\frac{r}{{A_{3} \Delta R}}} \right)^{2} } \right]} \right\},$$

where, *A*_1_, *A*_2_, *A*_3_, and *∆R* were set to be 5.5 × 10^9^ W/cm^2^, 0.85, 0.5, and 15 µm, respectively. To compare these calculated profiles with the conventional Gaussian laser spatial profile condition, we also carried out a simulation with parameters *A*_1_, *A*_2_, and ∆*R* in Eq. (5) set to 4.3 × 10^9^ W/cm^2^, 0.0, and 15 µm, respectively.
